# Clinical-Radiomics Nomogram Based on Contrast-Enhanced Ultrasound for Preoperative Prediction of Cervical Lymph Node Metastasis in Papillary Thyroid Carcinoma

**DOI:** 10.3390/cancers15051613

**Published:** 2023-03-05

**Authors:** Liqing Jiang, Zijian Zhang, Shiyan Guo, Yongfeng Zhao, Ping Zhou

**Affiliations:** 1 Department of Ultrasound, The Third Xiangya Hospital, Central South University, Changsha 410013, China; 198302053@csu.edu.cn (L.J.); 228302077@csu.edu.cn (S.G.); redscv@csu.edu.cn (Y.Z.); 2 Xiangya Lung Cancer Center, Xiangya Hospital, Central South University, Changsha 410008, China; wanzzj@csu.edu.cn; 3 Department of Radiation Oncology, Xiangya Hospital, Central South University, Changsha 410008, China; 4 National Clinical Research Center for Geriatric Disorders, Xiangya Hospital, Changsha 410008, China

**Keywords:** papillary thyroid carcinoma, cervical lymph node metastasis, radiomics, contrast-enhanced ultrasound, nomogram

## Abstract

**Simple Summary:**

Improving the precision of preoperative LNM assessment is crucial for determining the scope of PTC surgery, reducing complications, and preventing recurrence. Few studies have applied radiomics analysis based on contrast-enhanced ultrasound (CEUS) to the prediction of LNM in PTC. Our study found that CEUS-based radiomics, as a promising quantitative analysis, provides incremental value to clinical prediction and management of LNM in PTC. In addition, the developed clinical-radiomics nomogram demonstrated promising value for predicting LNM. It may be an effective, noninvasive tool for preoperative prediction of LNM in clinical use.

**Abstract:**

This study aimed to establish a new clinical-radiomics nomogram based on ultrasound (US) for cervical lymph node metastasis (LNM) in papillary thyroid carcinoma (PTC). We collected 211 patients with PTC between June 2018 and April 2020, then we randomly divided these patients into the training set (*n* = 148) and the validation set (*n* = 63). 837 radiomics features were extracted from B-mode ultrasound (BMUS) images and contrast-enhanced ultrasound (CEUS) images. The maximum relevance minimum redundancy (mRMR) algorithm, least absolute shrinkage and selection operator (LASSO) algorithm, and backward stepwise logistic regression (LR) were applied to select key features and establish a radiomics score (Radscore), including BMUS Radscore and CEUS Radscore. The clinical model and clinical-radiomics model were established using the univariate analysis and multivariate backward stepwise LR. The clinical-radiomics model was finally presented as a clinical-radiomics nomogram, the performance of which was evaluated by the receiver operating characteristic curves, Hosmer–Lemeshow test, calibration curves, and decision curve analysis (DCA). The results show that the clinical-radiomics nomogram was constructed by four predictors, including gender, age, US-reported LNM, and CEUS Radscore. The clinical-radiomics nomogram performed well in both the training set (AUC = 0.820) and the validation set (AUC = 0.814). The Hosmer–Lemeshow test and the calibration curves demonstrated good calibration. The DCA showed that the clinical-radiomics nomogram had satisfactory clinical utility. The clinical-radiomics nomogram constructed by CEUS Radscore and key clinical features can be used as an effective tool for individualized prediction of cervical LNM in PTC.

## 1. Introduction

Thyroid cancer (TC) ranked ninth among the incidence of human malignancies worldwide [1], and papillary thyroid carcinoma (PTC) is the most common pathological type among TC, accounting for 80–90% of cases [2]. PTC often has a good prognosis and a low mortality rate [3]; however, some PTCs exhibit cervical lymph nodes metastasis (LNM), which increases the risk of local recurrence and decreases overall patient survival [4,5]. Surgery is the main treatment for PTC, but because there are no effective solutions for predicting cervical LNM before surgery, it is still debatable whether to do prophylactic cervical lymph node (LN) dissection in patients with PTC. Although some experts recommend prophylactic cervical LN dissection for PTC patients [6,7], other studies suggest that this procedure may increase the risk of postoperative complications rather than considerably improve survival [8,9]. Therefore, it is crucial to improve the precision of preoperative LNM assessment to determine the scope of PTC surgery and enhance patient survival.

Preoperatively noninvasive assessment of cervical LNM is challenging. Ultrasound (US) is commonly used and plays an important role [10], as radiologists can determine the presence of cervical LNM by observing the sonographic features of bilateral cervical LNs. However, the detection rate of metastatic LNs by conventional US is not satisfactory, especially in the central region [7]. Given this, some scholars have targeted their studies on the US characteristics of primary tumors. Liu et al. reported that intratumoral microcalcification and parenchyma microcalcification were independent risk factors for LNM of PTC [11]. Zhan et al. found that cervical LNM was associated with high or equal enhancement at the peak time of CEUS, heterogeneous enhancement, and PTC size [12]. However, no validated and accepted model has been established, and US evaluation is susceptible to differences in the professional experience of radiologists.

Radiomics offers the potential for noninvasively precise diagnosis and treatment by extracting high-throughput quantitative information from medical images to create models that forecast intrinsic heterogeneity to support clinical decision-making [13]. Several scholars have successfully applied the radiomics approach from the primary tumor to predict LNM in malignant tumors, such as cervical cancer [14], pancreatic ductal adenocarcinoma [15], and laryngeal squamous cell carcinoma [16]. Several studies have not only confirmed the feasibility of primary tumor-based radiomics analysis for predicting cervical LNM in patients with PTC but also have combined the ultrasound radiomics signature and clinical data to construct robust predictive models for predicting LNM. The radiomics in these studies are mostly based on B-mode ultrasound (BMUS) and ultrasound elastography [17,18,19]. Few studies applied CEUS-based radiomics from primary tumors to the prediction of LNM in PTC, and it is not clear whether integrating CEUS-based radiomics with clinical risk factors enhances the ability to predict LNM in PTC.

The nomogram is based on a multivariate regression analysis that integrates multiple predictors, then graphically depicts the numerical relationship between the specific disease and risk factors, and finally intuitively provides the numerical probability of an outcome event through a scoring system [20,21,22]. Therefore, we aim to construct and validate a nomogram model combining the use of CEUS radiomics and clinical data to provide tools for the non-invasive prediction of cervical LNM in PTC for clinicians, thereby achieving the goal of individualized medicine.

## 2. Materials and Methods

### 2.1. Study Population

This study was approved by the ethics committee at the Third Xiangya Hospital. Written informed consent was waived owing to the retrospective nature of this study. We retrospectively collected patients with thyroid nodules who underwent preoperative ultrasound examination at the ultrasound department of our hospital between June 2018 and April 2020. Inclusion criteria: (1) postoperative pathology confirmed PTC; (2) treated with thyroidectomy and cervical LN dissection; (3) primary and solitary thyroid carcinoma; (4) no preoperative anticancer treatment; (5) ultrasound examinations were performed within two weeks before surgery. Exclusion criteria: (1) the lesion displayed incompletely in the US image due to excessive size; (2) poor image quality. A total of 211 patients were finally included. According to postoperative pathology, the patients were divided into the LNM group and the non-LNM group.

### 2.2. Image Acquisition and Clinicoradiological Characteristics Collection

Ultrasonography was performed using a GE LOGIQ E9 color Doppler ultrasonic instrument with a 9L linear array probe (2–9 MHz). The US physician first performed a BMUS examination of the thyroid gland, and saved the BMUS image of the largest long-axis section of the lesion, then switched to real-time CEUS mode. Next, the US physician asked the patient to breathe calmly and tried to keep the observation section unchanged. The contrast agent used was the SonoVue (Bracco, Milan, Italy). The patient received a bolus injection of 2.4 mL contrast agent through the antecubital vein, followed immediately by 5 mL of normal saline. The US physician observed the dynamic perfusion process of the lesion continuously and stored the dynamic images. A frame of the image at the peak time of CEUS was selected to store. Finally, two images of each nodule (BMUS image and CEUS image) were exported in Dicom format.

The conclusion suggestive of “LNM” in the US report was considered to be US-reported LN status positive. The conclusions of “undetectable LN”, “reactive hyperplastic lymph nodes” and “visible LN“ in the absence of metastasis were considered to be US-reported LN status negative. According to the 2015 American Thyroid Association (ATA) guidelines [23], the suspicious US signs suggestive of cervical LNM included round shape (aspect ratio > 0.5), calcifications, cystic changes, hyperechogenicity, and peripheral blood flow signals. One or more LNs that met one or more of the five criteria would be considered positive.

The following ultrasound features were recorded: primary site (left lobe, right lobe, isthmus), location (sub-capsular, intra-thyroidal), tumor size, echogenicity (iso/hyperechoic, hypoechoic, marked hypoechoic), aspect ratio (>1, ≤1), margin (smooth, ill-defined, irregular), calcification (absent or present), enhancement pattern (hypo-enhancement, iso-enhancement, hyper-enhancement). Demographic characteristics including gender and age were collected from the medical records.

### 2.3. Image Segmentation and Feature Extraction

ITK-SNAP software (open source software; http://www.itksnap.org, accessed on 7 August 2020) was used to segment the nodules, and the region of interest (ROI) was outlined along the contour of the targeted lesion. To assess interobserver reproducibility, 30 cases were randomly selected from all cases, and the images were segmented by two US physicians (reader1 and reader2), respectively. One US physician (reader1) performed all image segmentation. Next, the radiomics plug-in of 3D-Slicer software was used to perform feature extraction of the thyroid nodules. Before extracting features, the images were normalized including resampling to a voxel size of 1 mm × 1 mm × 1 mm, setting the bin width parameter in 3D-Slicer at 25 HU to discretize the voxel intensity. 837 radiomics features were extracted from each BMUS image and each CEUS image, respectively, and feature categories included first-order statistics, gray level dependence matrix (GLDM), gray level co-occurrence matrix (GLCM), gray level run length matrix (GLRLM), gray level size zone matrix (GLSZM) and neighborhood gray tone difference matrix (NGTDM).

### 2.4. Feature Selection and Radiomics Score Construction

We divided the 211 patients into a training set (*n* = 148) and validation set (*n* = 63) by 7:3 stratified random sampling method, and then radiomics features in the training and validation sets were z-score normalized according to the mean and standard deviation of the training set.

The process of radiomics feature selection and radiomics score construction is as follows. First, we calculated the interclass correlation coefficient (ICC) based on the radiomics features extracted after image segmentation by the two US physicians, and highly reproducible (ICC > 0.75) features were retained. Then the redundant and irrelevant features were removed using the minimum redundancy maximum correlation (mRMR) algorithm, and the best top 30 features from each image were selected. Next, the radiomics features associated with LNM were obtained by using the least absolute shrinkage and selection operator (LASSO) algorithm. Finally, a backward stepwise logistic regression (LR) with Akaike information criterion (AIC) was used to select the features constituting the logistic regression model, and the model score was the radiomics score (Radscore). According to the above process, we obtained BMUS Radscore and CEUS Radscore, respectively. Then a Mann–Whitney U test was used to assess the association of the Radscore with LNM. 

### 2.5. Development of the Clinical Model and the Clinical-Radiomics Nomogram

We constructed the model based on the training set and subsequently applied the model in the validation set to test its performance. In the training set, first, we performed a univariate analysis of clinical parameters (including demographic parameters and ultrasound features) and two Radscores. Stepwise multivariate LR analysis was then performed to develop a clinical model using clinical risk factors with *p*-value < 0.05 in the univariate analysis as candidate predictors.

Clinical risk factors and two Radscores were introduced into multivariate LR to build the clinical-radiomics combined model. A backward stepwise selection process with the AIC as the stopping rule was performed. A nomogram based on the clinical-radiomics model was drawn to visualize the logistic regression model for individualized assessment of patients’ risk of cervical LNM.

### 2.6. Model Validation

We plotted the receiver operating characteristic (ROC) curves and evaluated the predictive ability of the clinical-radiomics nomogram by the area under the ROC curve (AUC). Comparisons between the clinical model and the clinical-radiomics model were made using the integrated discrimination improvement (IDI) index.

We used the Hosmer–Lemeshow test and calibration curve to assess the calibration performance of the clinical-radiomics nomogram and used decision curve analysis (DCA) to assess the clinical utility of the clinical-radiomics nomogram by estimating the net benefit of the training set at each threshold probability.

### 2.7. Statistical Analysis

R software and associated packages were used for statistical analyses. Quantitative data were presented as mean ± standard deviation or median ± interquartile ranges. The t-test or Mann–Whitney U test was used to compare the differences in the measurement data between the two groups, and the Chi-square test or Fisher’s exact test was used to compare the differences in the enumeration data between the two groups. The difference between the two groups was statistically significant with *p* < 0.05.

## 3. Results

### 3.1. Clinicoradiological Characteristics

**Figure 1:**
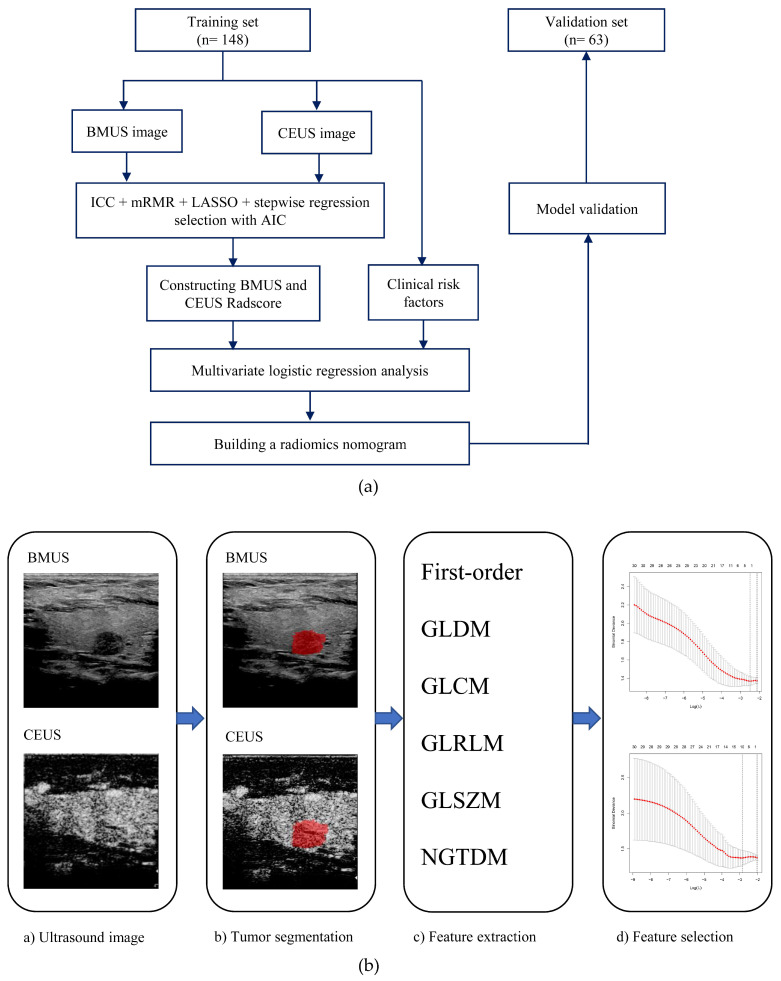
(**a**) Study flowchart of clinical-radiomics nomogram modeling for the LNM prediction in patients with PTC; (**b**) Radiomics workflow. BMUS, B-mode ultrasound; CEUS, contrast-enhanced ultrasound; ICC, interclass correlation coefficient; mRMR, minimum redundancy maximum relevance; LASSO, least absolute shrinkage and selection operator; AIC, Akaike information criterion; Radscore, radiomics score.

The study flowchart and radiomics workflow are reported in Figure 1. This study included 211 patients with solitary PTC, 88 of whom had positive cervical LNM results and 123 of whom had negative cervical LNM results. The patients were randomly divided in a 7:3 ratio, with 148 cases allocated to the training set and 63 cases allocated to the validation set, and the positive rate of cervical LNM was 39.9% and 46.0% in the training and validation sets, respectively, with no statistically significant difference (*p* = 0.406). Patients in the training and validation sets are listed by their clinical features in Table 1. Between the training and validation sets, there was no statistically significant difference in the clinical characteristics of patients (*p* > 0.05 for all), indicating that the baseline data were comparable for both sets. Table 2 shows the univariate analysis results between cervical LNM and candidate variables in the two groups. Age, tumor size, and US-reported LN status were associated with LNM in both the training and validation set (*p* < 0.05). Primary site, echogenicity, margin, microcalcification, and enhancement patterns were not associated with LNM in the training or validation set (*p* > 0.05). In the training set, males were more likely to have LNM (*p* < 0.05); but gender was not associated with LNM (*p* > 0.05) in the validation set. In the validation set, sub-capsular location was more likely to have LNM (*p* < 0.05); but tumor location was not associated with LNM (*p* > 0.05) in the training set.

### 3.2. Radiomics Score Building

**Figure 2:**
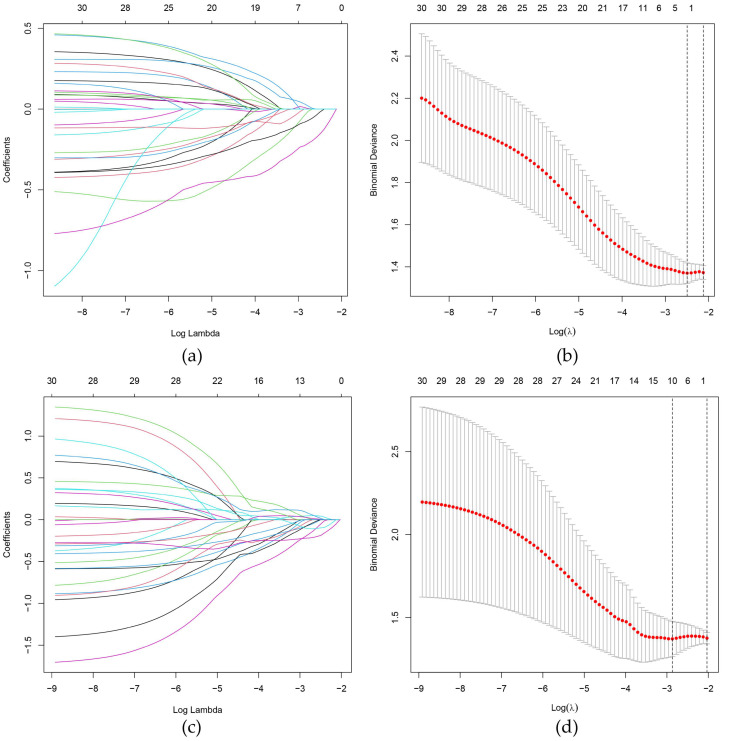
Selection of radiomics features using the least absolute shrinkage and selection operator (LASSO) algorithm in the training set. (**a**,**c**) LASSO coefficient profiles of the BMUS (**a**) and CEUS (**c**) features. (**b**,**d**) The 10-fold cross-validation and the minimum criteria process were used to generate the optimal penalization coefficient lambda (λ) in the BMUS and CEUS LASSO models. Dotted vertical lines are drawn by using the minimum criteria and 1 standard error of the minimum criteria. As a result, λ values of 0.08255607 and 0.05676918 were selected for the BMUS (**b**) and CEUS (**d**) features, respectively.

After removing the less stable features with ICC ≤ 0.75, 768 and 775 features were kept from the BMUS and CEUS images of each patient, respectively; 30 features were retained in each image by the mRMR algorithm. After the LASSO regression (Figure 2), 2 features from BMUS images and 10 features from CEUS images were selected. After the backward stepwise logistic regression analysis, 1 radiomics feature from the BMUS and 5 radiomics features from CEUS images were found to associate with LNM and used to construct the BMUS radiomics score and CEUS radiomics score, respectively. The formulas for BMUS Radscore and CEUS Radscore were as follows:

BMUS Radscore = −0.4535 − 0.5901 × wavelet.HLH_glszm_ZonePercentage(1)

CEUS Radscore = − 0.5624 − 0.5753 × wavelet.LHL_glcm_Idn − 0.4804 × wavelet.LHL_gldm_DependenceVariance + 0.3809 × wavelet.HHH_firstorder_Median + 0.5590 × original_glszm_SizeZoneNonUniformity − 0.9770 × wavelet.LHH_glrlm_GrayLevelNonUniformityNormalized(2)

### 3.3. Model Building and Validation

**Figure 3:**
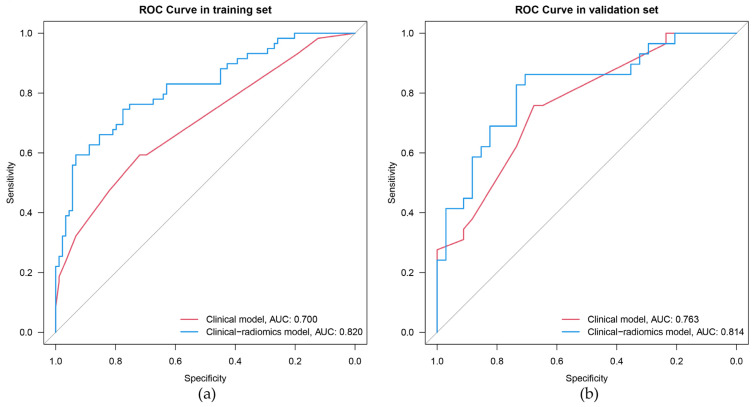
The ROC curves of the clinical model and clinical-radiomics model. ROC, receiver operating characteristic. (**a**) The training set, (**b**) the validation set.

The univariate results showed significant differences (*p* < 0.05) in age, gender, tumor size, and US-reported LN status between the LNM positive and negative groups in the training set (Table 2). After backward stepwise multivariate logistic regression analysis, age < 55 years and US-reported LN status positive were still identified to be significant factors (*p* < 0.05) for LNM (Table 3). The AUCs for the clinical model were 0.700 (0.617–0.784) and 0.763 (0.650–0.877) in the training and validation set, respectively (Figure 3). 

**Figure 4:**
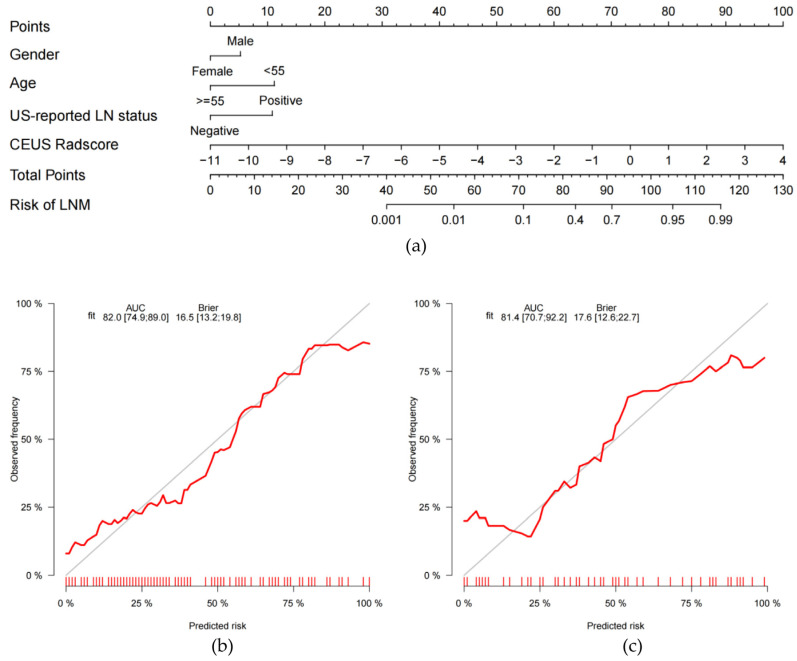
Developed radiomic nomogram based on the clinical-radiomics model for the assessment of cervical LNM in PTC patients (**a**). Calibration curves of the clinical-radiomics nomogram in the training set (**b**) and validation set (**c**).

**Figure 5:**
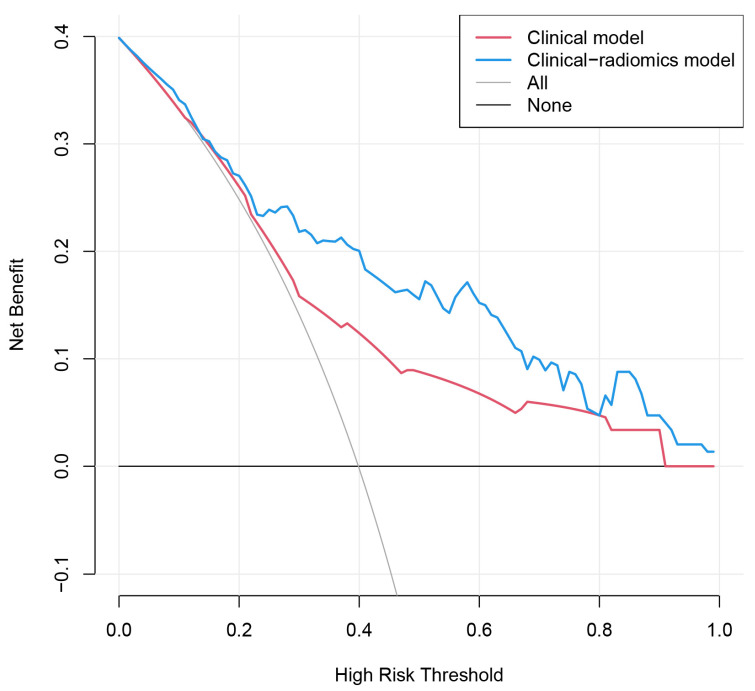
Decision curve of clinical model and clinical-radiomics model. The decision curve analysis (DCA) measures the net benefit (*y*-axis) versus the model’s high-risk threshold (*x*-axis) for different models. The black horizontal line manifests no patients with LNM (none) and the grey line manifests all patients with LNM (all). The decision curves indicate that the net benefit of the clinical-radiomics nomogram was higher than the clinical model across the majority of the range of reasonable risk thresholds.

Six factors, namely, age, gender, tumor size, US-reported LN status, BMUS Radscore, and CEUS Radscore were introduced into stepwise multivariate logistic regression. As a result, the clinical-radiomics combined model was constructed based on gender, age, US-reported LN status, and CEUS Radscore (Table 3). In the combined model, age (OR, 0.18; 95%CI, 0.05–0.70), US-reported LN status (OR, 5.16; 95%CI, 1.40–18.98), and CEUS Radscore (OR, 2.75; 95%CI, 1.79–4.23) were independently associated with LNM. The AUCs for the combined model were 0.820 (0.749–0.890) and 0.814 (0.707–0.922) in the training and validation set, respectively (Figure 3). Then we compared the clinical model and the clinical-radiomics model [IDI = 15.42% (9.15–21.69%), *p* < 0.001 in the training set; IDI = 8.59% (0.91–16.26%), *p* = 0.028 in the validation set], a notable improvement in discrimination was seen in the clinical-radiomics model. This might mean that the addition of CEUS Radiomics improved LNM risk discrimination beyond the clinical model. We visualized the clinical-radiomics model using a clinical-radiomics nomogram (Figure 4A). The calibration curves (Figure 4B,C) and the Hosmer–Lemeshow test revealed that there was no significant difference between the probability predicted by the clinical-radiomics nomogram and actual probabilities (the Hosmer–Lemeshow test: *p*-value = 0.569 in the training set; *p*-value = 0.558 in the validation set). The DCA (Figure 5) showed that a treatment plan based on the clinical-radiomics nomogram might be more beneficial than either the treat-all-patients strategy or the treat-none strategy, and the net benefit of the clinical-radiomics nomogram was higher than the clinical model across the majority of the range of threshold probabilities.

## 4. Discussion

Currently, surgery is the main treatment for PTC. However, it is controversial whether total thyroidectomy and prophylactic lymph node dissection can provide substantial benefits for patients with PTC. Furthermore, surgical demolitive interventions may be accompanied by more severe complications, such as recurrent laryngeal nerve paralysis, cervical hematoma, and hypoparathyroidism [24,25]. Therefore, preoperatively prognostic markers to assess the risk of cervical LNM in PTC are of great significance to effectively avoid overdiagnosis and improve prognosis. To this end, Vincenzo Marotta et al. found that germline VEGF-A single nucleotide polymorphisms (SNPs) were stable and accessible prognostic markers for DTC (Differentiated Thyroid Cancer; PTC accounts for 85% of DTC [23]) obtained by peripheral blood testing, and constitute promising tools to enhance prognostic stratification of DTC [26]. In addition, Zhang et al. analyzed the BRAFV600E mutation from thyroid nodule samples collected by Fine-Needle Aspiration (FNA) biopsy and found that BRAFV600E mutation was an independent prognostic marker of central cervical LNM in PTC [27]. Unlike the aforementioned markers, radiomics is a non-invasive, time-saving, and cost-effective prognostic marker which was confirmed by many recent studies [13,28,29,30].

In the current study, we developed and validated a clinical-radiomics nomogram that combines key clinical risk factors and CEUS radiomics features for the individualized prediction of LNM in PTC. Compared with the clinical model, the clinical-radiomics nomogram had the better diagnostic efficacy for predicting LNM, with AUCs of 0.820 and 0.814, in the training set and validation set, respectively. Thus, our study suggests that clinical-radiomics nomogram can be used to assess the risk of LNM for PTC patients preoperatively and non-invasively, and provide a reference for individualized treatment planning.

According to the TNM staging system of the AJCC 8th edition [23], 55 years was used as the cut-off value for the age of patients with PTC in this study. Both univariate and multivariate analyses showed that age was significantly and negatively associated with LNM, and young age was an independent risk factor for cervical LNM in patients with PTC. This is consistent with previous findings [11,31]. Therefore, during the preoperative US examination, it was crucial to carefully examine the LN status in young patients with PTC. Male PTC patients had a higher likelihood of LNM than female PTC patients [32,33,34], which was also confirmed by our findings. This may be related to sex hormones [35]. After selection with the backward stepwise method, gender was kept in the final model. Both univariate and multivariate analyses showed that US-reported LN status was significantly associated with LNM, so US-reported LN status was included in the final prediction model. This is similar to other studies [18,19], showing that US-reported LN status is an important part of the preoperative prediction. However, US has limitations in the assessment of cervical LNM. Central cervical LNM was easily missed due to its deep location and the thyroid gland that overlies it; some meta-analyses have shown that the sensitivity of ultrasound for the assessment of central cervical LNM is less than 35% [7,36]. In addition, US diagnosis is based on visual qualitative judgments that were constrained by US physicians’ experience differences. Therefore, complementary indicators are needed for a more efficient diagnosis.

In recent years, radiomics is one of the hot research topics in medical imaging. Radiomics analysis can overcome the possible strong subjectivity of traditional medical image interpretation and convert medical imaging data into quantitative biomarkers through innovative computational methods [37]. US-based radiomics techniques have developed rapidly and have been applied to the differential diagnosis of tumors and the assessment of tumor aggressiveness, including malignant parotid gland lesions [38], breast cancer [39,40], and renal cell carcinoma [41]. With regard to PTC, Enock Adjei Agyekum et al. [42] reported that the radiomics model based on preoperative US images provided promising results in assessing cervical LNM in patients with PTC. Some studies have explored the further application of multimodal US radiomics [19,43,44]. Our preliminary study found that radiomics analysis of BMUS and CEUS images has good diagnostic efficacy for discriminating thyroid nodules, and the diagnostic efficacy of the BMUS + CEUS radiomics model is superior, suggesting the potential application value of multimodal radiomics in identifying benign and malignant thyroid nodules [45]. CEUS can help for preoperative prediction of LNM in PTC by intravenously injecting a blood pool contrast agent to show tissue microperfusion [5]. In this study, we explored the use of combined BMUS and CEUS images for radiomics analysis in predicting LNM of PTC. It is noticeable that in univariate analysis, BMUS Radscore and CEUS Radscore were significantly associated with LNM, but BMUS Radscore did not enter into the final clinical-radiomics model, which is similar to the results of Jiang et al. [19], showing that the BMUS Radscore was excluded due to its insufficient predictive power for LNM. We discovered that in the final multivariate stepwise logistic regression, the superior discriminatory power of CEUS Radscore weakened the weight of the BMUS Radscore.

Previous studies have found that the CEUS enhancement pattern of tumors can help predict LNM and that hyper- or iso-enhancement can be an independent risk factor for LNM [12,46]. The enhancement pattern of CEUS in our study is not related to LNM, probably due to the different data set with the small sample size of high enhancement in our study; in addition, enhancement intensity, as a qualitative characteristic judged by the naked eye, involves a certain degree of subjectivity. This does not affect our inspiring finding that quantitative radiomics analysis based on the CEUS image is strongly associated with LNM. In contrast to visual inspection of enhancement intensity and homogeneity, radiomics may be able to quantitatively decode important information about the heterogeneity of tumor microcirculation, which is associated with intratumoral perfusion, vascular permeability, and angiogenesis [47,48,49]. The CEUS Radscore included five radiomics features, and 80% of the selected radiomics features were wavelet-based features. The wavelet transform can reveal the hidden features of medical images at multiple scales [50,51], amplify the heterogeneous information of target tumor texture features, and enhance the discriminative ability [52]. We also noticed that most selected CEUS radiomics features characterize the spatial distribution of lesion voxels, proving that PTCs with higher vascular heterogeneity are prone to exhibit aggressive biological behavior. These radiomics features, which are hard to identify with the naked eye, have the potential to be non-invasive biomarkers for the preoperative prediction of cervical LN status in PTC.

Finally, we combined the radiomics score and key preoperative clinical features to create a clinical-radiomics model, and for clinical application, a nomogram was created as a visualization of the logistic regression model. The AUC and clinical benefit of the clinical-radiomics nomogram were higher than using the clinical model. Combining key clinical features with CEUS Radscore resulted in a significant improvement in IDI, demonstrating the incremental value of CEUS-based radiomics for preoperative clinical prediction of LN status.

The limitations of our study should be acknowledged. 1: This study was a retrospective single-center study, so a prospective multicenter study with a large sample size is needed for further improvement before practical application. 2: Our radiomics analysis of the CEUS image was based on a single-frame image due to technological limitations, and much information might be missed compared to the analysis of the entire perfusion process. Further research is needed into image processing and feature extraction of the dynamic image. 3: The radiomics analysis in this study was based on the images of the primary tumors, and there are still few studies that establish a radiomics model based on LN sonograms for LNM prediction in PTC patients. Future research is needed to determine the feasibility and predictive value of radiomics analysis based on LN sonograms or a combination of the primary tumor and LN images.

## 5. Conclusions

In conclusion, we constructed a clinical-radiomics nomogram incorporating CEUS Radscore and key clinical features. It demonstrated favorable predictive ability for LNM in patients with PTC and can be used as an effective tool for individualized prediction of cervical LNM in PTC.

## Figures and Tables

**Table 1:** Baseline patient characteristics in training and validation sets.

Characteristic	Training Set (*n* = 148)	Validation Set (*n* = 63)	*p*-Value
Lymph node metastasis			0.406
Negative	89 (60.1)	34 (54.0)	
Positive	59 (39.9)	29 (46.0)	
Age			0.531
<55 years	125 (84.5)	51 (81.0)	
≥55 years	23 (15.5)	12 (19.0)	
Gender			0.495
Female	111 (75.0)	50 (79.4)	
Male	37 (25.0)	13 (20.6)	
Primary site			0.299
Left lobe	61 (41.2)	33 (52.4)	
Right lobe	78 (52.7)	26 (41.3)	
Isthmus	9 (6.1)	4 (6.3)	
Tumor location			0.980
Intra-thyroidal	35 (23.6)	15 (23.8)	
Sub-capsular	113 (76.4)	48 (76.2)	
Tumor size			0.106
≤10 mm	106 (71.6)	38 (60.3)	
>10 mm	42 (28.4)	25 (39.7)	
Echogenicity			0.583
iso/hyperechoic	7 (4.7)	3 (4.8)	
hypoechoic	58 (39.2)	20 (31.7)	
marked hypoechoic	83 (56.1)	40 (63.5)	
Aspect ratio > 1			0.757
Absent	93 (62.8)	41 (65.1)	
Present	55 (37.2)	22 (34.9)	
Margin			0.579
Smooth	7 (4.7)	3 (4.8)	
Ill-defined	12 (8.1)	8 (12.7)	
Irregular	129 (87.2)	52 (82.5)	
Microcalcification			0.571
Absent	39 (26.4)	19 (30.2)	
Present	109 (73.6)	44 (69.8)	
Enhancement pattern			0.329
Hyper-enhancement	7 (4.7)	1 (1.6)	
Iso-enhancement	34 (23.0)	11 (17.5)	
Hypo-enhancement	107 (72.3)	51 (81.0)	
US-reported LN status			0.062
Negative	130 (87.8)	49 (77.8)	
Positive	18 (12.2)	14 (22.2)	
BMUS Radscore,			0.662
Median (Interquartile range)	−0.40 (−0.71, −0.07)	−0.32 (−0.84, 0.10)	
CEUS Radscore,			0.185
Median (Interquartile range)	−0.54 (−1.20, 0.29)	−0.37 (−0.87, 0.36)	

**Table 2:** Associations between the lymph node metastasis and patient characteristics in the training and validation sets.

Characteristic	Training Set			Validation Set		
	LNM−	LNM+	*p*-Value	LNM−	LNM+	*p*-Value
Age			0.017			0.023
<55 years	70 (78.7)	55 (93.2)		24 (70.6)	27 (93.1)	
≥55 years	19 (21.3)	4 (6.8)		10 (29.4)	2 (6.9)	
Gender			0.042			0.060
Female	72 (80.9)	39 (66.1)		30 (88.2)	20 (69.0)	
Male	17 (19.1)	20 (33.9)		4 (11.8)	9 (31.0)	
Primary site			0.642			0.401
Left lobe	34 (38.2)	27 (45.8)		17 (50.0)	16 (55.2)	
Right lobe	49 (55.1)	29 (49.2)		16 (47.1)	10 (34.5)	
Isthmus	6 (6.7)	3 (5.1)		1 (2.9)	3 (10.3)	
Tumor location			0.118			0.020
Intra-thyroidal	25 (28.1)	10 (16.9)		12 (35.3)	3 (10.3)	
Sub-capsular	64 (71.9)	49 (83.1)		22 (64.7)	26 (89.7)	
Tumor size			0.002			<0.001
>10 mm	72 (80.9)	34 (57.6)		27 (79.4)	11 (37.9)	
≤10 mm	17 (19.1)	25 (42.4)		7 (20.6)	18 (62.1)	
Echogenicity			0.409			0.497
iso/hyperechoic	5 (5.6)	2 (3.4)		1 (2.9)	2 (6.9)	
hypoechoic	31 (34.8)	27 (45.8)		13 (38.2)	7 (24.1)	
marked hypoechoic	53 (59.6)	30 (50.8)		20 (58.8)	20 (69.0)	
Aspect ratio > 1			0.309			0.029
Absent	53 (59.6)	40 (67.8)		18 (52.9)	23 (79.3)	
Present	36 (40.4)	19 (32.2)		16 (47.1)	6 (20.7)	
Margin			1.000			0.146
Smooth	4 (4.5)	3 (5.1)		1 (2.9)	2 (6.9)	
Ill-defined	7 (7.9)	5 (8.5)		2 (5.9)	6 (20.7)	
Irregular	78 (87.6)	51 (86.4)		31 (91.2)	21 (72.4)	
Microcalcification			0.083			0.336
Absent	28 (31.5)	11 (18.6)		12 (35.3)	7 (24.1)	
Present	61 (68.5)	48 (81.4)		22 (64.7)	22 (75.9)	
Enhancement pattern			0.155			0.860
Hyper-enhancement	2 (2.2)	5 (8.5)		0 (0.0)	1 (3.4)	
Iso-enhancement	23 (25.8)	11 (18.6)		6 (17.6)	5 (17.2)	
Hypo-enhancement	64 (71.9)	43 (72.9)		28 (82.4)	23 (79.3)	
US-reported LN status			<0.001			0.031
Negative	85 (95.5)	45 (76.3)		30 (88.2)	19 (65.5)	
Positive	4 (4.5)	14 (23.7)		4 (11.8)	10 (34.5)	
BMUS Radscore			0.001			0.004
Median (Interquartile range)	−0.51 (−0.85, −0.21)	−0.25 (−0.50, 0.04)		−0.53 (−1.00, −0.16)	−0.02 (−0.52, 0.28)	
CEUS Radscore			<0.001			0.002
Median (Interquartile range)	−0.89 (−1.71, −0.28)	0.12 (−0.54, 0.66)		−0.66 (−1.18, −0.07)	0.10 (−0.37, 0.75)	

**Table 3:** Clinical model and clinical-radiomics model based on stepwise multivariate analyses for prediction of LNM.

Characteristics	Odds Ratio (95%CI)	*p*-Value
Clinical model		
Gender (male vs. female)	2.18 (0.95, 5.00)	0.067
Age (≥55 years vs. <55 years)	0.30 (0.09, 0.96)	0.042
Tumor size (>10 mm vs. ≤10 mm)	2.22 (1.00, 4.95)	0.051
US-reported LN status (positive vs. negative)	4.86 (1.40, 16.83)	0.013
Clinical-radiomics model		
Gender (male vs. female)	2.22 (0.86, 5.74)	0.100
Age (≥55 years vs. <55 years)	0.18 (0.05, 0.70)	0.013
US-reported LN status (positive vs. negative)	5.16 (1.40, 18.98)	0.014
CEUS Radscore	2.75 (1.79, 4.23)	<0.001

Abbreviations: CI, confidence interval.

## Data Availability

The original contributions presented in the study are included in the article; further inquiries can be directed to the corresponding author.
